# Epileptic Seizure Detection Using Machine Learning: A Systematic Review and Meta-Analysis

**DOI:** 10.3390/brainsci15060634

**Published:** 2025-06-12

**Authors:** Lin Bai, Gerhard Litscher, Xiaoning Li

**Affiliations:** 1 Heilongjiang University of Traditional Chinese Medicine, Harbin 150040, China; bl18846925895@126.com; 2 Swiss University of Traditional Chinese Medicine, SWISS TCM UNI, High-Tech Acupuncture and Digital Chinese Medicine, 5330 Bad Zurzach, Switzerland; 3 Department of Acupuncture, The Second Affiliated Hospital of Heilongjiang University of Traditional Chinese Medicine, Harbin 150006, China

**Keywords:** artificial intelligence, deep learning, machine learning, epilepsy, seizure detection, EEG, meta-analysis

## Abstract

Background/Objectives: Epileptic seizures significantly impact patients’ lives due to their unpredictability, making early and accurate detection crucial for effective treatment. Machine learning (ML) models based on electroencephalogram (EEG) signals have been explored for automated seizure detection. This meta-analysis reviews the performance of ML models in seizure detection and analyzes factors such as the model type (deep learning vs. traditional ML), data preprocessing methods, and dataset types. Aim: This study aims to provide an evidence-based foundation for the future development of intelligent tools by evaluating the performance of ML models in detecting epileptic seizures through a meta-analysis. Methods: A systematic search of multiple databases up to April 2025 identified 60 studies and 93 datasets. The pooled sensitivity, specificity, and area under the curve (AUC) were calculated using Stata 17.0. Subgroup analyses were performed to identify sources of heterogeneity. Publication bias was assessed using Deek’s test and funnel plots. Results: The pooled sensitivity, specificity, and AUC were 0.96 (95% CI 0.95–0.97), 0.97 (95% CI 0.96–0.98), and 0.99 (95% CI 0.98–1.00), respectively, indicating a good performance of ML in seizure detection. Subgroup analyses revealed that the model type, data preprocessing methods, and dataset type contributed to heterogeneity. Conclusions: ML shows a strong potential for EEG-based seizure detection. Imaging devices integrating ML may serve as effective tools for early epilepsy diagnosis. However, larger, multicenter clinical studies are needed to validate these algorithms and enhance their interpretability, safety, and applicability in real-world clinical settings.

## 1. Introduction

Epilepsy is a chronic neurological disorder characterized by sudden, recurrent, and unprovoked seizures and is caused by abnormal synchronous neuronal discharges in the brain [1]. It affects over 70 million people worldwide [2], covering all age groups and contributing significantly to the global disease burden across all age groups [3]. Clinically, seizures can manifest as a loss of consciousness, motor convulsions, or subtle behavioral changes, often resulting in falls, injuries, or even death. Importantly, approximately 70% of epilepsy patients can become seizure-free with a prompt and accurate diagnosis and treatment [4]. Hence, prompt and reliable seizure detection is critical to reducing morbidity and improving the quality of life for patients.

Traditionally, the diagnosis of epilepsy involves a combination of clinical assessments and the use of a range of diagnostic tools including electroencephalography (EEG), magnetic resonance imaging (MRI), computed tomography (CT), positron emission tomography (PET), and magnetoencephalography (MEG) [5]. Among these, EEG remains the gold standard for epilepsy diagnosis due to its high temporal resolution, non-invasiveness, and cost-effectiveness [6]. However, the diagnostic value of EEG largely depends on visual interpretations by experienced neurologists, psychiatrists, or neurosurgeons and may vary across epilepsy types (e.g., focal vs. generalized, symptomatic vs. idiopathic). It is also important to recognize that EEG abnormalities may not be evident in interictal recordings, especially in early- or subtle-stage epilepsy, further complicating diagnosis. Additionally, the epileptiform features of an EEG, such as spikes, sharp waves, or spike–wave complexes, require expert judgment, and no single criterion is universally adopted [7,8,9,10].

To address these limitations, automated seizure detection using machine learning (ML) has gained significant attention. ML can process vast amounts of EEG data to detect subtle or transient patterns not easily visible to the human eye. ML models typically extract handcrafted or learned features from time, frequency, time–frequency, or nonlinear domains [11,12] and classify signals using algorithms such as support vector machines (SVMs), k-nearest neighbors (KNNs), decision trees (DTs), and neural networks.

Furthermore, recent advances in deep learning (DL), a subfield of ML, have enabled end-to-end seizure detection from raw EEG, reducing the need for manual feature extraction and exhibiting good prospects in real-time applications. Despite this, DL models often lack interpretability and transparency, which poses challenges for their clinical integration where trust and interpretability are essential.

The field has grown rapidly, yet there is considerable heterogeneity in model designs, evaluation methods, datasets used (e.g., CHB-MIT, Bonn, Freiburg), and reported outcomes. Most studies rely on internal validation using limited or publicly available datasets, raising concerns about overfitting, dataset biases, and the limited generalizability to diverse clinical populations. Moreover, few studies include comparisons with clinical experts or a prospective validation in real-world settings.

Several narrative reviews have been conducted, but few involved a quantitative meta-analysis with subgroup analyses and a bias assessment. Thus, a comprehensive synthesis of the current evidence is still lacking. To fill this gap, we conducted a systematic review and meta-analysis to evaluate the diagnostic performance of ML-based algorithms for seizures using EEG data.

Specifically, we aimed to (1) assess the pooled sensitivity, specificity, and area under the curve (AUC) of traditional ML and DL models; (2) investigate the sources of heterogeneity from the perspective of the model type, data preprocessing method, and dataset type; (3) evaluate the publication bias and methodological quality of included studies using the AI-specific QUADAS-AI tool; and (4) identify limitations and research gaps relevant to clinical translation.

Our findings will provide a rigorous, up-to-date overview of the ML performance in EEG-based seizure detection and inform future directions for research and clinical implementation.

## 2. Materials and Methods

### 2.1. Registration

This study followed the PRISMA guidelines [13] and registered with PROSPERO (CRD42024588410). No informed consent or ethical approval was required.

### 2.2. Search Strategy

A search was conducted in PubMed, Embase, Web of Science, IEEE, and Cochrane Library for cohort, case–control, nested case–control, and cross-sectional studies on the performance of ML models in the detection of epileptic seizures up to 15 January 2025. A second search was conducted before finalizing the manuscript. The search terms consisted of “deep learning”, “machine learning”, “epilepsy”, “cohort study”, “case–control study”, “nested case–control study”, and “cross-sectional study” (Supplementary Table S1). Letters, conference abstracts, scientific reports, and narrative reviews were excluded. The reference lists of identified studies and narrative reviews were manually searched for potentially missing studies. Two trained reviewers (L.B. and Y.L.) were independently responsible for the search, and any discrepancy was settled by discussion with a third reviewer (X.L.) and consensus.

### 2.3. Eligibility Criteria

#### 2.3.1. Inclusion Criteria

PICOS principle: (1) Participant: Patients diagnosed with epilepsy or suspected of epilepsy, with no restriction on age, sex, or race. (2) Intervention: Traditional ML and deep learning (DL) models, such as support vector machine, random forest, decision tree, and convolutional/recurrent neural network. (3) Comparison: Traditional methods for seizure detection, such as clinical presentation analysis and manual EEG analysis. (4) Outcome: Sensitivity (SE), specificity (SP), and accuracy. (5) Study design: Cohort, case–control, nested case–control, and cross-sectional studies. We only included peer-reviewed academic papers rather than comments, conference abstracts, and unpublished gray literature.

#### 2.3.2. Exclusion Criteria

(1) Duplicate publications or studies with similar data; (2) studies focusing only on risk factors for seizures and assessing only the predictive accuracy of single factors; (3) reviews, guidelines, systematic reviews, meta-analyses, case reports, expert experience, conference reports, and basic experiments; and (4) studies for which full text was not available or the data required for meta-analysis were not extractable.

### 2.4. Study Screening and Data Extraction

Spreadsheets were created to extract the study characteristics and the data on diagnostic performance, including first author, year of publication, Reference Standard, type of internal validation, presence or absence of external validation, model types (traditional ML/DL), and source of datasets. Contingency tables were used to extract binary diagnostic data (true-positive, false-positive, true-negative, and false-negative). If multiple contingency tables were provided for the same or different ML algorithms in one study, they were assumed to be mutually independent. We would contact the authors by e-mail to obtain, as far as possible, unpublished or missing data. Two trained reviewers (L.B. and Y.L.) were independently responsible for study screening, data extraction, and cross-checking. Any discrepancy was settled by discussion with a third reviewer (X.L.) and consensus.

### 2.5. Quality Assessment

Two reviewers (L.B. and Y.L.) assessed the study quality by using the Quality Assessment of Diagnostic Accuracy Studies-Artificial Intelligence (QUADAS-AI) [14] from four domains of risk of bias (RoB) and three of applicability (Table S2). Combining QUADAS-2 [15] and QUADAS-C [16], QUADAS-AI is specifically applied to assess RoB and applicability in ML-related studies. The four domains (Patient Selection, Index Test, Reference Standard, and Flow and Timing) each contain specific questions (“Yes”, “No”, or “Uncertain” as “Low”, “High”, or “Unclear” RoB). A study was considered to have a low RoB if all key questions were answered “Yes” in each domain. Any answer as “No” indicated potential RoB, and then the level of risk was assessed according to established guidelines. An answer as “Unclear” indicated no sufficient information to make a definitive judgment. Any discrepancy was resolved by discussion with a third reviewer (X.L.) and consensus. Finally, a RoB diagram was generated.

### 2.6. Data Analysis

The diagnostic performance of the ML algorithms was assessed using forest plots and Summary Receiver Operating Characteristic (SROC) curves. Relative mean SE, SP, and AUC were estimated, with pooled SE and SP calculated using bivariate mixed-effects models along with 95% confidence intervals (CIs). Further meta-analyses reported the optimal accuracy of multiple ML algorithms based on contingency tables. Heterogeneity among studies was evaluated using the I^2^ statistic, and its potential sources were investigated through subgroup analyses. Publication bias was assessed using funnel plots. ROC curves were generated by combining diagnostic effect sizes and their variances or standard errors for each study, allowing pooled SE and SP calculations at various cutoff points. A *p*-value < 0.05 was considered statistically significant. Subgroup analyses were performed based on the model type (DL or traditional ML), data preprocessing method (band-pass filtering and Discrete Wavelet Transform [DWT]), and dataset type (CHB-MIT, Bonn, Siena Scalp, Freiburg, and others). Meta-analyses were conducted only when at least three original studies were included per subgroup. All analyses were conducted using Stata 17.

## 3. Results

### 3.1. Search Results

**Figure 1:**
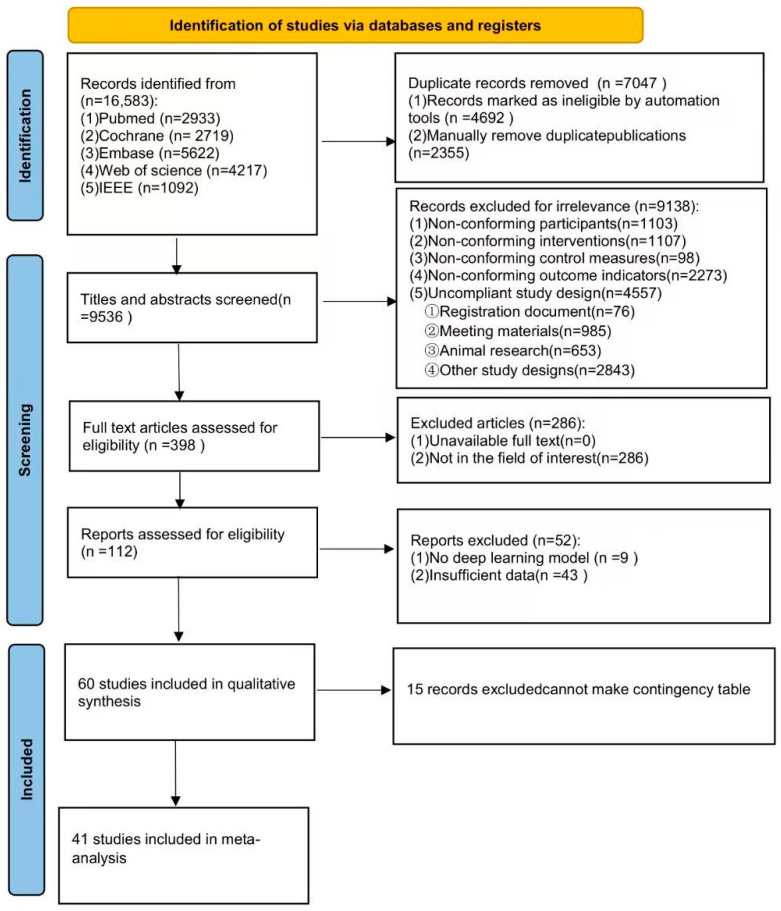
The PRISMA (Preferred Reporting Items for Systematic Reviews and Meta-Analyses) flowchart for this study.

We initially retrieved 16,583 potentially relevant studies. After removing 7047 duplicates, 9138 studies were excluded based on the title and abstract screening. The full texts of the remaining articles were then assessed for eligibility, leading to the exclusion of 286 studies due to irrelevant study fields, 9 studies for not reporting deep learning (DL) models, and 43 studies due to overlapping data. Ultimately, a few dozen studies [17,18,19,20,21,22,23,24,25,26,27,28,29,30,31,32,33,34,35,36,37,38,39,40,41,42,43,44,45,46,47,48,49,50,51,52,53,54,55,56,57,58,59,60,61,62,63,64,65,66,67,68,69,70,71,72,73,74] met the inclusion criteria and were included in the meta-analysis. The detailed study selection process is illustrated in the PRISMA flowchart (Figure 1).

### 3.2. Study Characteristics

The characteristics of the 58 included studies [17,18,19,20,21,22,23,24,25,26,27,28,29,30,31,32,33,34,35,36,37,38,39,40,41,42,43,44,45,46,47,48,49,50,51,52,53,54,55,56,57,58,59,60,61,62,63,64,65,66,67,68,69,70,71,72,73,74], along with the baseline characteristics of the patient populations, are summarized in Supplementary Tables S1–S3. Among them, 49 studies focused on deep learning (DL) approaches [17,18,19,20,21,22,23,24,25,26,27,28,29,30,31,32,33,34,35,36,37,38,39,40,41,42,43,44,45,46,47,48,49,50,51,52,53,54,55,56,57,58,59,60,61,62,63,64,65], while 9 studies applied classical machine learning (ML) methods [66,67,68,69,70,71,72,73,74].

All studies [17,18,19,20,21,22,23,24,25,26,27,28,29,30,31,32,33,34,35,36,37,38,39,40,41,42,43,44,45,46,47,48,49,50,51,52,53,54,55,56,57,58,59,60,61,62,63,64,65,66,67,68,69,70,71,72,73,74] utilized retrospective data obtained from publicly available open-access databases; no study employed prospectively collected datasets. Moreover, all analyses were based on internal validations, with no application of external validations using out-of-sample data.

In terms of the study design, 24 studies were conducted as multicenter analyses [17,19,20,22,25,30,33,36,41,43,45,46,48,50,51,52,58,59,61,62,66,69,71,73], while 34 studies were performed at a single center [18,21,23,24,26,27,28,29,31,32,34,35,37,38,39,40,42,44,47,49,53,54,55,56,57,60,63,64,65,67,68,70,72,74].

In terms of validation strategies, a 10-fold cross-validation was the most frequently used method, applied in 28 studies [18,21,22,23,25,29,30,31,35,36,37,40,41,42,43,46,47,50,51,53,55,57,58,59,60,62,64,68]. A 5-fold cross-validation was adopted in twelve studies [17,19,28,33,38,44,52,54,56,66,69], and the leave-one-out method was used in seven studies [24,26,34,41,49,54,61].

Regarding datasets, a variety of EEG sources were utilized:

- The CHB-MIT dataset was used in 35 studies [17,18,19,20,21,22,23,27,28,29,31,33,35,39,40,41,43,45,46,48,49,50,51,54,57,58,61,63,64,65,66,68,69,72,73];
- The Bonn dataset was used in 26 studies [17,18,20,22,25,26,30,33,36,37,38,41,42,44,45,46,50,52,57,58,59,60,61,62,71,73];
- The SWEC-ETHZ dataset was used in seven studies [24,30,34,43,47,51,70];
- The Freiburg dataset was used in seven studies [32,36,53,56,59,71,74];
- The Siena Scalp dataset was used in three studies [22,66,69];
- Other datasets (including proprietary or combined datasets) were used in seven studies [19,25,46,48,52,55,67].

### 3.3. Overall Performance of ML Algorithms

**Figure 2:**
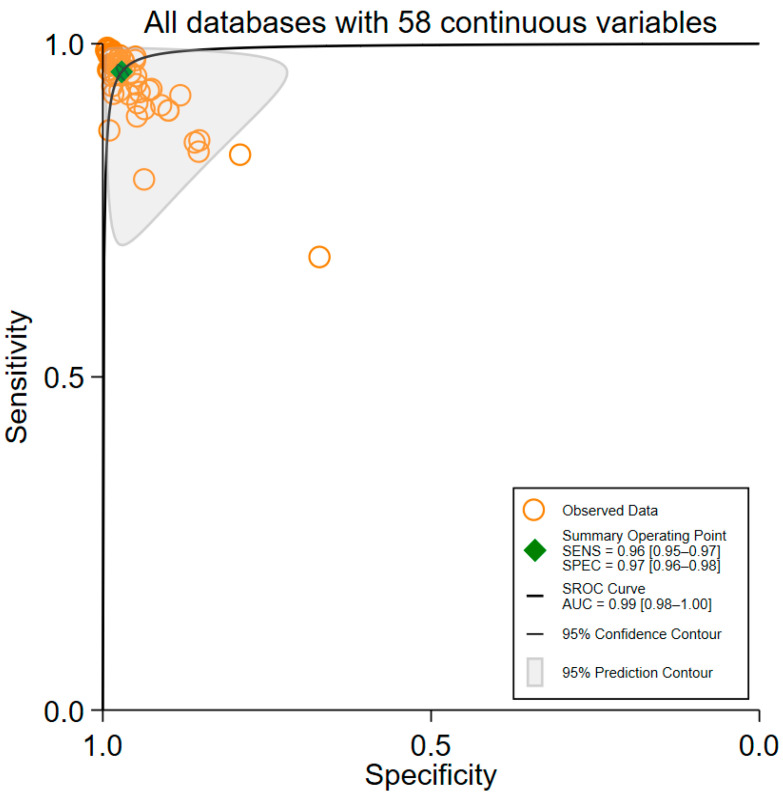
This figure presents the Summary Receiver Operating Characteristic (SROC) curves derived from 58 contingency tables across 60 included studies. The ROC curves illustrate the trade-off between sensitivity and specificity for the pooled results of all machine learning (ML) algorithms evaluated. The high area under the curve indicates an excellent overall diagnostic accuracy. The pooled sensitivity and specificity suggest that ML models robustly detect epileptic seizures with minimal false-positives and false-negatives. The tight confidence intervals reflect consistency across studies despite the underlying heterogeneity. This figure summarizes the collective performance of diverse ML approaches applied to EEG signals for seizure detection, highlighting their potential clinical utility.

The majority of the 41 studies [17,18,19,23,25,26,27,29,31,32,33,35,36,37,38,39,40,42,44,45,46,50,51,53,55,57,59,60,61,63,64,65,66,67,68,69,70,71,72,73,74] reported the diagnostic performance of more than one ML algorithm. As a result, sufficient data were available to construct 58 contingency tables, which served as the basis for generating SROC curves (Figure 2). For all ML algorithms evaluated, the pooled SP was 0.97 (95% CI: 0.96–0.98), and the pooled SE was 0.96 (95% CI: 0.95–0.97). The AUC was 0.99 (95% CI: 0.98–1.00), indicating the excellent overall diagnostic accuracy of the ML algorithms.

### 3.4. Subgroup Analyses

We conducted three independent meta-analyses to explore the diagnostic performance of ML algorithms in relation to the (I) model type, (II) data preprocessing method, and (III) dataset type.

**Figure 3:**
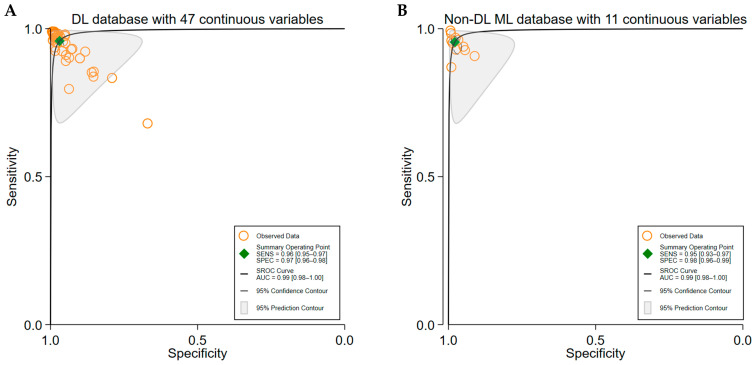
Pooled diagnostic performance comparison between deep learning (DL) and traditional machine learning (ML) algorithms for seizure detection using EEG data. Panel (**A**) shows the Summary Receiver Operating Characteristic (SROC) curve for DL algorithms, based on data from 49 studies encompassing 47 contingency tables. The DL models achieved a pooled sensitivity and specificity, indicating an outstanding accuracy in seizure detection. Panel (**B**) presents the SROC curve for traditional ML algorithms from 7 studies with 11 contingency tables. These models showed a slightly lower but still excellent performance. The comparison highlights that both DL and ML algorithms provide high diagnostic accuracy for the EEG-based seizure detection. Both approaches are effective.

I. Model Type:

Deep learning (DL) models were reported in 49 studies [17,18,19,20,21,22,23,24,25,26,27,28,29,30,31,32,33,34,35,36,37,38,39,40,41,42,43,44,45,46,47,48,49,50,51,52,53,54,55,56,57,58,59,60,61,62,63,64,65], yielding 47 contingency tables. The pooled sensitivity (SE) and specificity (SP) were 0.96 (95% CI: 0.95–0.97) and 0.97 (95% CI: 0.96–0.98), respectively, with an area under the curve (AUC) of 0.99 (95% CI: 0.98–1.00) (Figure 3A). Classical ML models were reported in seven studies [38,55,60,61,65,66,72], generating 11 contingency tables, with a pooled SE and SP of 0.95 (95% CI: 0.93–0.97) and 0.98 (95% CI: 0.96–0.99), respectively, and an AUC of 0.99 (95% CI: 0.98–1.00) (Figure 3B).

**Figure 4:**
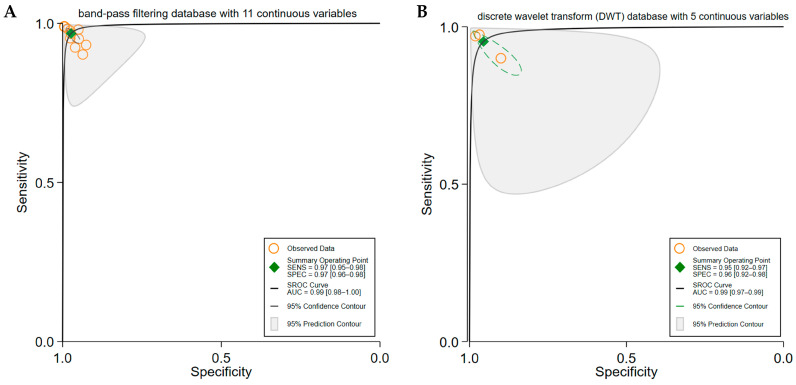
The pooled diagnostic performance of seizure detection models based on different EEG data preprocessing methods. Panel (**A**) displays the Summary Receiver Operating Characteristic (SROC) curve for models using band-pass filtering as the preprocessing technique, compiled from 7 studies with 11 contingency tables. Panel (**B**) illustrates the SROC curve for models using Discrete Wavelet Transform (DWT) for feature extraction, based on 3 studies with 5 contingency tables. These showed slightly lower performances.

II. Data Preprocessing Method (Feature Type):

- Band-pass filtering was used in 11 contingency tables from seven studies [17,27,31,33,63,73,74], resulting in a pooled SE of 0.97 (95% CI: 0.95–0.98), SP of 0.97 (95% CI: 0.96–0.98), and AUC of 0.99 (95% CI: 0.98–1.00) (95% CI: 0.99–1.00) (Figure 4A).
- The Discrete Wavelet Transform (DWT) was used in five contingency tables from three studies [37,39,68], with a pooled SE of 0.95 (95% CI: 0.92–0.97), a SP of 0.96 (95% CI: 0.92–0.98), and an AUC of 0.99 (95% CI: 0.97–0.99) (Figure 4B).

Additionally, EEG signals from the Freiburg, Siena Scalp, and other datasets were incorporated across preprocessing methods.

**Figure 5:**
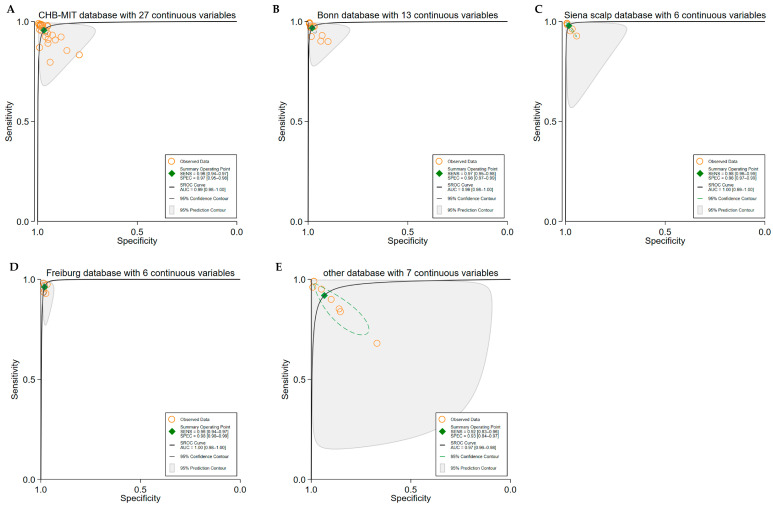
The pooled performance based on different dataset types. (**A**) ROC curves for CHB-MIT (24 studies, 27 contingency tables), (**B**) ROC curves for Bonn (13 studies, 13 contingency tables), (**C**) ROC curves for Siena Scalp (3 studies, 6 contingency tables), (**D**) ROC curves for Freiburg (6 studies, 6 contingency tables), and (**E**) ROC curves for other datasets (7 studies, 11 contingency tables). The pooled diagnostic performance based on different EEG dataset types. Panel (**A**) shows the ROC curves for models developed using the CHB-MIT dataset, which includes 24 studies with 27 contingency tables; Panel (**B**) presents the performance on the Bonn dataset, based on 13 studies and 13 contingency tables; Panel (**C**) displays the results for the Siena Scalp dataset (3 studies, 6 contingency tables); and Panel (**D**) shows findings from the Freiburg dataset (6 studies, 6 contingency tables). Finally, Panel (**E**) summarizes results from other datasets across 7 studies and 11 contingency tables. These results demonstrate the consistently high diagnostic performance of seizure detection models across multiple EEG datasets, though some variability in confidence intervals reflects differences in dataset characteristics.

III. Dataset Type:

- CHB-MIT was used in 24 studies [17,18,19,23,25,27,31,32,33,35,40,42,44,50,55,60,63,65,66,67,69,71,73,74], contributing 27 contingency tables. The pooled SE and SP were 0.96 (95% CI: 0.94–0.97) and 0.97 (95% CI: 0.95–0.98), respectively, with an AUC of 0.99 (95% CI: 0.98–1.00) (Figure 5A).
- Bonn was used in 12 studies [17,19,27,37,38,46,50,51,57,59,64,68], across 12 contingency tables, with a pooled SE and SP of 0.97 (95% CI: 0.95–0.98) and 0.98 (95% CI: 0.97–0.99) and an AUC of 0.99 (95% CI: 0.98–1.00) (Figure 5B).
- Siena Scalp was employed in three studies [63,65,66], resulting in six contingency tables, with an SE and SP of 0.98 (95% CI: 0.96–0.99) and 0.98 (95% CI: 0.97–0.99) and an AUC of 1.00 (95% CI: 0.99–1.00) (Figure 5C).
- The Freiburg dataset appeared in six studies [26,39,40,68,70,72], contributing six contingency tables, with an SE of 0.96 (95% CI: 0.94–0.97), SP of 0.98 (95% CI: 0.98–0.99), and AUC of 1.00 (95% CI: 0.98–1.00) (Figure 5D).
- Other datasets were reported in seven studies [31,32,39,45,53,61], encompassing 11 contingency tables, with a pooled SE and SP of 0.92 (95% CI: 0.83–0.96) and 0.93 (95% CI: 0.84–0.97), and an AUC of 0.97 (95% CI: 0.96–0.98) (Figure 5E).

### 3.5. Heterogeneity Analysis

It was observed in all included studies that ML was useful for detecting seizures based on EEG, but extreme heterogeneity was found. The I^2^ value was 99.96% for the SE and 1.00 for the SP (Figure S1). The source of heterogeneity was explored by subgroup analyses:

I. Model type: DL (SE: I^2^ = 99.98%, SP: I^2^ = 1.00) (Figure S2A) and traditional ML (SE: I^2^ = 98.85%, SP: I^2^ = 99.99%) (Figure S2B).

II. Data preprocessing method (feature type): Band-pass filtering (SE: I2 = 98.89%, SP: I^2^ = 99.98%) (Figure S3A) and DWT (SE: I^2^ = 98.06%, SP: I^2^ = 99.99%) (Figure S3B).

Dataset type: CHB-MIT (SE: I^2^ = 99.82%, SP: I^2^ = 1.00) (Figure S4A), Bonn (SE: I^2^ = 96.27%, SP: I^2^ = 97.31%) (Figure S4B), Siena Scalp (SE: I^2^ = 99.56%, SP: I^2^ = 99.97%) (Figure S4C), Freiburg (SE: I^2^ = 98.83%, SP: I^2^ = 99.96%) (Figure S4D), and other datasets (SE: I^2^ = 99.99%, SP: I^2^ = 1.00) (Figure S4E).

### 3.6. Quality Assessment

**Figure 6:**
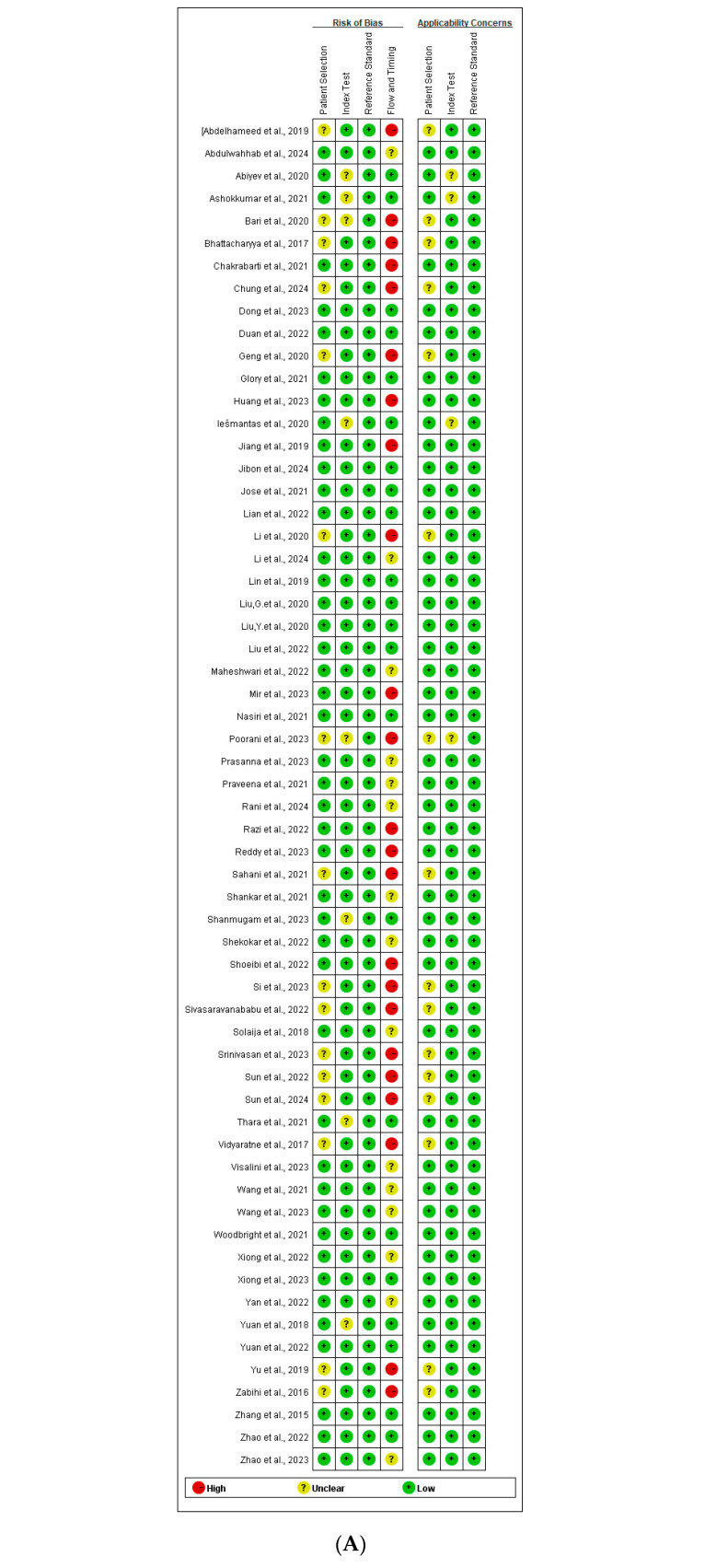

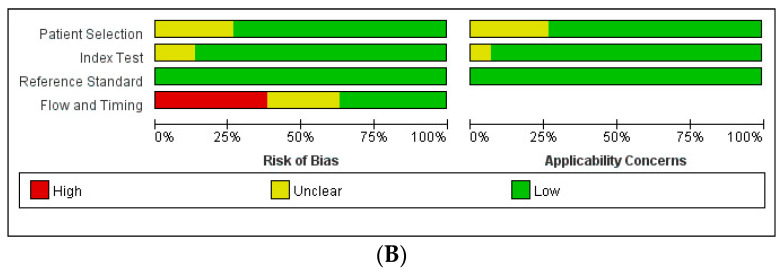
The risk of bias (RoB) assessment across included studies using QUADAS-AI. (**A**) An individual study-level RoB diagram summarizing the judgments for each QUADAS-AI domain: the Patient Selection, Index Test, Reference Standard, and Flow and Timing. Colors represent a low (green), high (red), or unclear (yellow) risk of bias for each domain per study. Notably, a high or unclear RoB was frequently observed in the domains of Patient Selection and Flow and Timing, indicating methodological concerns such as inappropriate exclusion criteria and an incomplete case inclusion [17,18,19,20,21,22,23,24,25,26,27,28,29,30,31,32,33,34,35,36,37,38,39,40,41,42,43,44,45,46,47,48,49,50,51,52,53,54,55,56,57,58,59,60,61,62,63,64,65,66,67,68,69,70,71,72,73,74]. (**B**) A pooled RoB summary presenting the overall proportion of studies falling into each risk category for the four QUADAS-AI domains. While the Reference Standard domain showed a predominantly low RoB due to the use of predefined thresholds, other domains demonstrated a considerable proportion of unclear or high RoB, particularly for the Index Test, due to insufficient reporting on blinding and validation methods. These findings highlight the need for more rigorously designed and transparently reported studies in ML-based EEG seizure detection.

The included studies [17,18,19,20,21,22,23,24,25,26,27,28,29,30,31,32,33,34,35,36,37,38,39,40,41,42,43,44,45,46,47,48,49,50,51,52,53,54,55,56,57,58,59,60,61,62,63,64,65,66,67,68,69,70,71,72,73,74] were evaluated for their methodological quality using the QUADAS-AI tool (Figure 6A,B). The quality assessment identified several areas of concern:

- Patient Selection: Fifteen studies [17,23,24,28,34,35,46,54,56,57,59,62,64,65,73] were rated as having an unclear risk of bias (RoB) due to potentially inappropriate patient exclusions.
- Index Test: Seven studies [25,28,44,55,57,58,63] were assessed as having an unclear RoB, primarily because they did not explicitly state whether the Index Test was interpreted without knowledge of the Reference Standard results. However, since all studies employed predefined thresholds, the RoB for the Reference Standard was rated as low across all studies.
- Flow and Timing:
  ○ Fifteen studies [17,23,24,28,34,35,46,54,56,57,59,62,64,65,73] exhibited a high RoB in this domain because not all eligible patients were included in the final analysis.
  ○ An additional twelve studies [18,21,22,27,38,39,43,45,47,67,69,72] were assigned an unclear RoB, as they lacked information about whether the same Reference Standard was applied or whether the time interval between the Index Test and Reference Standard was appropriate.

In terms of applicability concerns:

- For Patient Selection, fifteen studies [17,23,24,28,34,35,46,54,56,57,59,62,64,65,73] were rated as either having high or unclear applicability concerns.
- For the Index Test, three studies [28,55,58] were rated as unclear.
- No studies were found to have applicability concerns related to the Reference Standard.

## 4. Discussion

In this study, 93 datasets [17,18,19,20,21,22,23,24,25,26,27,28,29,30,31,32,33,34,35,36,37,38,39,40,41,42,43,44,45,46,47,48,49,50,51,52,53,54,55,56,57,58,59,60,61,62,63,64,65,66,67,68,69,70,71,72,73,74] were meta-analyzed, making it the largest investigation to date assessing the performance of ML algorithms for seizure detection using EEG data. The results demonstrated that ML exhibits great potential for EEG-based seizure detection, with a high SE and SP, thereby laying an important foundation for clinical applications. As a non-invasive technique, ML can be widely deployed to alleviate healthcare resource shortages, increase seizure detection rates, and enable earlier diagnoses. In this way, patients can undergo prompt treatment, improving survival rates and prognosis [75].

However, to facilitate the adoption of AI in medicine for real-world clinical practice and standardized diagnostic workflows, more high-quality prospective studies on the performance of ML algorithms against experienced clinicians in actual clinical settings are necessary. Future research applying ML to portable devices with real-time monitoring capabilities holds great promise for enhancing epilepsy detection and early interventions, advancing personalized medicine, and further improving patient outcomes.

Following a systematic study search, three other systematic reviews and meta-analyses on ML algorithms in EEG were identified. Two of these conducted only systematic reviews without additional subgroup, sensitivity, or publication bias analyses and focused on different fields and study populations, limiting a direct comparison with our study [76,77]. Zou et al. [78] meta-analyzed the ML model performance in monitoring pediatric epileptic seizures but were restricted by small sample sizes and single-population data. Our study included patients of all ages, sexes, and races and conducted subgroup analyses by the model type (DL vs. traditional ML), data preprocessing method, and dataset type, which may inform future research. Moreover, we employed the novel AI-specific quality assessment tool QUADAS-AI [14], an extension of QUADAS-2 [15] and QUADAS-C [16], which incorporates AI-relevant domains such as the training, validation/test datasets, patient selection diversity, and reference standard appropriateness.

We also highlighted the distinctions and advantages between DL and ML. ML algorithms, due to their interpretability and transparency, are more likely to gain clinicians’ trust and be integrated into clinical decision-making, enhancing personalized care [79]. However, ML often requires manual segmentation and feature extraction, which can introduce heterogeneity and bias [80]. In contrast, DL excels at automated image and signal analysis and outperforms ML in real-time seizure monitoring [31]. Nevertheless, DL models lack interpretability, which may restrict clinical acceptance [47]. Future efforts should be conducted to develop interpretable AI models to improve transparency and trust, address the “black box” issue, and facilitate clinical adoption [81].

Variations in the data preprocessing method and dataset type contribute to heterogeneity and restrict the generalizability of ML models. Many included studies [17,18,19,20,21,22,23,25,26,27,28,29,30,31,32,33,34,35,36,37,38,40,41,42,43,44,45,46,47,48,49,50,51,52,53,54,55,56,57,58,59,60,61,62,63,64,65,66,67,68,69,70,71,72,73,74] relied on small, homogeneous datasets underrepresenting minority groups. Data preprocessing biases further obscure the ML performance across datasets. To mitigate these biases, more studies encompassing diverse populations from different countries, races, and socioeconomic backgrounds are needed to better represent ML performance in real-world clinical settings.

Several limitations should be noted: First, funnel plots revealed a potential publication bias (Supplementary Figure S5), likely due to a scarcity of prospective studies and underreporting of negative results. Second, most data were derived from publicly available open-access databases lacking detailed participant characterization, complicating adjustments for confounders and emphasizing the need for prospective clinical data. Third, the predominance of positive results in ML studies may bias the dataset. We encourage researchers to publish null or negative findings to better approximate true effect sizes [82]. Finally, this meta-analysis relied solely on internal validation, which may overestimate the diagnostic accuracy. External validation using larger, multicenter datasets across diverse populations is critical to confirming the robustness and generalizability of ML models prior to clinical deployment [83]. Incorporating clinical variables into EEG characterization may also improve diagnostic accuracy.

## 5. Conclusions

ML demonstrates great potential for EEG-based seizure detection. The integration of ML into imaging and monitoring devices can become a powerful tool for an early diagnosis and timely intervention in epilepsy. However, to advance clinical translation, large-scale, prospective, and multicenter studies are urgently needed to validate and refine these algorithms. Enhancing model interpretability, ensuring patient safety, and building clinician trust are essential for the widespread adoption of ML. Furthermore, validation across diverse at-risk populations and real-world clinical settings is critical to confirming their effectiveness and generalizability.

## Supplementary Materials

The following supporting information can be downloaded at https://www.mdpi.com/article/10.3390/brainsci15060634/s1, Table S1: Study design and basic demographics. Table S2: Indicators, algorithms and data sources. Table S3: Design and basic demographics. Figure S1: Forest plots for all studies. Figure S2: Forest maps of different model classifications (DL (A), ML (B)). Figure S3: Different (feature type) data preprocessing methods (band-pass filtering (A) or DWT forest graph (B)). Figure S4: Forest maps of different data sets (CHB-MIT (A), Bonn (B), Siena scalp (C), Freiburg database (D), other (E). Figure S5: Publication bias.

## Abbreviations

The following abbreviations are used in this manuscript:

AUC	Area Under the Curve
CI	Confidence Interval
EEG	Electroencephalogram
ML	Machine Learning
ROC	Receiver Operating Characteristic
ROB	Risk of Bias
SROC	Summary Receiver Operating Characteristic
