# Supplementary material for: Epileptic Seizure Detection Using Machine Learning: A Systematic Review and Meta-Analysis
Source: Brain Sci. 2025 Jun 12;15(6):634. doi: 10.3390/brainsci15060634 (PMC12190198; doi:10.3390/brainsci15060634)
Supplement: Supplementary file 1 [file brainsci-15-00634-s001.zip › Table S1.pdf]

Table S1. Study design and basic demographics.

Author [ref], year	Reference standard	Type of internal validation	External validation	Model classification (ML[a]/DL[b])	Publicly available
Sun et al. [57], 2024	Consist Label	5-fold cross-validation	No	DL	Yes
Li et al. [34], 2024	Consist Label	10-fold cross-validation	No	DL	Yes
Jibon et al. [31], 2024	Consist label	5-fold cross-validation	No	DL	Yes
Chung et al. [23], 2024	Consist Label	NR	No	DL	Yes
Abdulwahhab et al. [18], 2024	Consist Label	4-fold cross-validation	No	DL	Yes
Rani et al. [46], 2024	Consist Label	5-fold cross-validation	No	DL	Yes
Zhao et al. [73], 2023	Consist Label	10-fold cross-validation	No	DL	Yes
Wang et al. [63], 2023	Consist Label	10-fold cross-validation	No	DL	Yes
Srinivasan et al. [56], 2023	Consist Label	10-fold cross-validation	No	DL	Yes
Si et al. [53], 2023	Consist Label	leave-one-out cross-validation	No	DL	Yes
Shanmugam et al. [51], 2023	Consist Label	10-fold cross-validation	No	DL	Yes
Reddy et al. [49], 2023	Expert Consensus	leave-one-out cross-validation	No	DL	Yes
Prasanna et al. [44], 2023	Consist Label	NR	No	DL	Yes
Poorani et al. [43], 2023	Consist Label	5-fold cross-validation	No	DL	Yes
Mir et al. [41], 2023	Expert Consensus	10-fold cross-validation	No	DL	Yes
Huang et al. [28], 2023	Expert Consensus	10-fold cross-validation	No	DL	Yes
Zhao et al. [74], 2022	Expert Consensus	10-fold cross-validation	No	DL	Yes
Yuan et al. [70], 2022	Consist Label	NR	No	DL	Yes
Yan et al. [67], 2022	Consist Label	5-fold cross-validation	No	DL	Yes
Sun et al. [58], 2022	Expert Consensus	leave-one-out cross-validation	No	DL	Yes
Sivasaravanababu et al. [54], 2022	Consist Label	10-fold cross-validation	No	DL	Yes
Shoeibi et al. [52], 2022	Consist Label	10-fold cross-validation	No	DL	Yes
Lian et al. [35], 2022	Consist Label	10-fold cross validation	No	DL	Yes
Duan et al. [25], 2022	Consist Label	10-fold cross-validation	No	DL	Yes

		leave-one-out cross-validation			
Maheshwari et al. [40], 2022	Consist Label	NR	No	DL	Yes
Woodbright et al. [64], 2021	Consist Label	10-fold cross-validation	No	DL	Yes
Wang et al. [62], 2021	Consist Label	NR	No	DL	Yes
Thara et al. [59], 2021	Consist Label	5-fold cross-validation	No	DL	Yes
Shankar et al. [50], 2021	Consist Label	NR	No	DL	Yes
Sahani et al. [48], 2021	Consist Label	10-fold cross-validation	No	DL	Yes
Praveena et al. [44], 2021	Consist Label	10-fold cross-validation	No	DL	Yes
Nasiri et al. [42], 2021	Consist Label	leave-one-out cross-validation	No	DL	Yes
Jose et al. [32], 2021	Expert Consensus	NR	No	DL	Yes
Chakrabarti et al. [22], 2021	Consist Label	10-fold cross-validation	No	DL	Yes
Glory et al. [27], 2021	Consist Label	10-fold cross-validation	No	DL	Yes
Liu et al. [37], 2020	Consist Label	5-fold cross-validation	No	DL	Yes
Liu et al. [39], 2020	Expert Consensus	10-fold cross-validation	No	DL	No
Li et al. [33], 2020	Consist Label	5-fold cross-validation/ leave-one-out cross-validation	No	DL	Yes
Iešmantas et al. [29], 2020	Consist Label	10-fold cross validation	No	DL	Yes
Geng et al. [26], 2020	Expert Consensus	5-fold cross-validation	No	DL	Yes
Bari et al. [20], 2020	Consist Label	10-fold cross validation	No	DL	Yes
Abiyev et al. [19], 2020	Consist Label	10-fold cross-validation	No	DL	Yes
Yu et al. [68], 2019	Expert Consensus	10-fold cross-validation	No	DL	Yes
Lin et al. [36], 2019	Consist Label	10-fold cross-validation	No	DL	Yes
Jiang et al. [30], 2019	Consist Label	leave-one-out cross-validation	No	DL	Yes
Abdelhameed et al. [17], 2019	Consist Label	10-fold cross-validation	No	DL	Yes
Yuan et al. [69], 2018	Expert Consensus	NR	No	DL	Yes
Bhattacharyya et al. [21], 2017	Consist Label	10-fold cross-validation	No	DL	Yes

Zabihi et al. [71], 2016	Consist Label	NR	No	DL	Yes
Xiong et al. [66], 2023	Consist Label	5-fold cross-validation	No	ML	Yes
Visalini et al. [61], 2023	Consist Label	NR	No	ML	No
Dong et al. [24], 2023	Consist Label	10-fold cross-validation	No	ML	Yes
Xiong et al. [65], 2022	Consist Label	5-fold cross-validation	No	ML	Yes
Razi et al. [47], 2022	Consist Label	NR	No	ML	Yes
Liu et al. [38], 2022	Expert Consensus	10-fold cross-validation	No	ML	Yes
Solaija et al. [55], 2018	Expert Consensus	27-fold cross-validation	No	ML	Yes
Vidyaratne et al. [60], 2017	Expert Consensus	5-fold cross-validation	No	ML	Yes
Zhang et al. [72], 2015	Expert Consensus	NR	No	ML	Yes

aML: machine learning.

bDL: deep learning.
