# Supplementary material for: Epileptic Seizure Detection Using Machine Learning: A Systematic Review and Meta-Analysis
Source: Brain Sci. 2025 Jun 12;15(6):634. doi: 10.3390/brainsci15060634 (PMC12190198; doi:10.3390/brainsci15060634)
Supplement: Supplementary file 1 [file brainsci-15-00634-s001.zip › Table S2.pdf]

Table S2. Indicators, algorithms and data sources.

Author [ref], year	Indicator defifinition		Algorithm		Data source			
	Device	Exclusion of poor-quality cases	Algorithm architecture	Transformer applied	Source of data	Number of cases for training/ test/ internal/ external	Data range	Open access data
Sun et al. [57], 2024	Single-channel<br>23-channel	Yes	MDFLN	Yes	①Children's Hospital Boston;<br>②Bonn University	CHB-MIT<br>training dataset :384052<br>validation dataset :128017<br>testing dataset :128017<br>Bonn<br>training dataset :4800<br>testing dataset :1600<br>validation dataset :1600	CHB-MIT:2010<br>Bonn :2001	Yes
Li et al. [34], 2024	Singal-channel	NR	EMD+MLP-pooling	Yes	Bonn University	training dataset :2400<br>testing dataset :800<br>validation dataset :800	2001	Yes
Jibon et al. [31], 2024	16-channel	NR	SGCN-Deep RNN	Yes	①Children's Hospital Boston;<br>②Temple University Hospital	CHB-MIT<br>training dataset :512069<br>testing dataset :128017<br>TUH<br>training dataset :336156<br>testing dataset :175680	CHB-MIT:2010<br>TUH :2007-2013	Yes
Chung et al. [23], 2024	18-, 4-, and Single-chan	Yes	NR	NR	Children's Hospital Boston;	training dataset :2353092<br>validation dataset :672312	2010	Yes

	nel					testing dataset :336156		
Abdulwahhab et al. [18], 2024	Single-channel<br>18-channel	NR	PCNN–LSTM	Yes	①Children's Hospital Boston;<br>②Bonn University	CHB-MIT<br>testing dataset :640086<br>Bonn<br>testing dataset :2000<br>training dataset :6000	CHB-MIT:2010<br>Bonn :2001	Yes
Rani et al. [46], 2024	20-channel<br>28-channel	NR	SSA-based LSTM	Yes	Bonn University	training dataset :6400<br>testing dataset :1600	2001	Yes
Zhao et al. [73], 2023	NR	NR	CNN + Transformer	Yes	Children's Hospital Boston	CHB-MIT<br>testing dataset :640086	2010	Yes
Wang et al. [63], 2023	Single-channel<br>23-channel	NR	Vietoris–Rips (VR) complex filtration model	No	①Children's Hospital Boston<br>② Siena Scalp dataset of the Unit of Neurology and Neurophysiology of the University<br>③Bonn University	segment-based classification (Experiment 1):<br>①CHB-MIT<br>training dataset :448060<br>testing dataset :192026<br>②Siena Scalp<br>training dataset :322560<br>testing dataset :138240<br>③Bonn<br>training dataset :3360<br>testing dataset :1440	CHB-MIT:2010<br>Siena Scalp :2020<br>Bonn :2001	Yes
Srinivasan et al. [56], 2023	3-channel	Yes	3D-DCAE+BiLSTM	Yes	Children's Hospital Boston	training dataset :576077<br>testing dataset :64009	2010	Yes
Si et al. [53], 2023	128-channel	Yes	CBAM	No	SWEC-ETHZ long-term iEEG dataset	training dataset :5720400<br>testing dataset :5720400	2016	Yes
Shanmugam et al. [51],	128-channel	NR	1D-CNN 1	NR	①Bonn University	Bonn	Bonn :2001	Yes

2023			LSTM		②Neurology and Sleep Centre (NSC).	training dataset :9600<br>testing dataset :2400<br>validation dataset :1200<br>NSC<br>training dataset :7200<br>testing dataset :1800<br>validation dataset :900	NSC :2016	
Reddy et al. [49], 2023	Single-chan nel	NR	CNN-FCM	No	Bonn University	training dataset :6400<br>testing dataset :1600	2001	No
Prasanna et al. [44], 2023	19-channel<br>26-channel	NR	BESD-Net	Yes	Children's Hospital Boston	training dataset :640086<br>testing dataset :640086	2010	Yes
Poorani et al. [43], 2023	23-channel	Yes	1D-CNN and CNN-LSTM	NR	Children's Hospital Boston	training dataset :1167488<br>testing dataset :291872	2010	Yes
Mir et al. [41], 2023	23-channel	NR	DCAE-ESD-Bi-LSTM	Yes	Children's Hospital Boston	training dataset :2000<br>testing dataset :500<br>validation dataset :500	2010	No
Huang et al. [28], 2023	Single-chan nel	NR	MRP-Net	Yes	①Bonn University<br>②the Sleep-Wake-Epilepsy Center (SWEC) of the Department of Neurology at Bern Hospital University.	Bonn<br>training dataset :7200<br>testing dataset :800<br>SWEC-ETHZ<br>training dataset :7516800<br>testing dataset :835200	Bonn :2001<br>SWEC-ETHZ :2016	Yes
Zhao et al. [74], 2022	23-channel<br>16-channel	NR	GAT	NR	Children's Hospital Boston	training dataset :512069<br>testing dataset :128017	2010	Yes
Yuan et al. [70], 2022	6-channel	NR	ProCRC-GN	Yes	the University Hospital of	training dataset :356400	2015	No

			MF		Freiburg	testing dataset :2005200		
Yan et al. [67], 2022	18-channel	NR	STONE	Yes	①Bonn University<br>②Children's Hospital Boston	CHB<br>training dataset :512069<br>validation dataset :128017<br>Bonn<br>training dataset :6400<br>validation dataset :1600	CHB-MIT:2010<br>Bonn :2001	Yes
Sun et al. [58], 2022	NR	Yes	NR	Yes	SWEC-ETHZ long-term iEEG dataset	training dataset :9234000<br>testing dataset :9234000	2016	Yes
Sivasaravanababu et al. [54], 2022	23-channel<br>18-channel	Yes	SB-LSTM	Yes	Children's Hospital Boston	training dataset :448060<br>testing dataset :64009<br>validation dataset :128017	2010	Yes
Shoeibi et al. [52], 2022	2-channel	NR	TQWT + Fuzzy Entropy Features Set + AE + ANFIS-BS	Yes	①Freiburg dataset<br>②Bonn University	Bonn<br>training dataset :1604160<br>test dataset :401040<br>Freiburg database<br>training dataset :1604160<br>testing dataset :401040	Freiburg:2015<br>Bonn :2001	Yes
Lian et al. [35], 2022	NR	NR	GCNN	NR	Children's Hospital Boston	training dataset :576077<br>testing dataset :64009	2010	Yes
Duan et al. [25], 2022	Single-channel	NR	NR	Yes	①Children's Hospital Boston;<br>②Bonn University	CHB<br>training dataset :640086<br>testing dataset :640086<br>validation dataset :71121<br>Bonn<br>training dataset :72000	CHB-MIT:2010<br>Bonn :2001	Yes

						testing dataset :8000		
Maheshwari et al. [40], 2022	23-channel	NR	NR	NR	Children's Hospital Boston	testing dataset :640086	2010	Yes
Woodbright et al. [64], 2021	NR	NR	NR	No	Bonn University	training dataset :7200<br>testing dataset :800	2001	Yes
Wang et al. [62], 2021	23-channel	NR	RS-DA strategy + S-1D-CNN	Yes	①Children's Hospital Boston;<br>②the SWEC-ETHZ iEEG dataset	CHB<br>training dataset :640086<br>testing dataset :640086<br>validation dataset :71121<br>SWEC-ETHZ<br>training dataset :9234000<br>testing dataset :9234000<br>validation dataset :923400	CHB-MIT:2010<br>SWEC-ETHZ :2016	Yes
Thara et al. [59], 2021	Single-channel	NR	LSTM_GAP	Yes	Bonn University	training dataset :46000<br>testing dataset :111500	2001	Yes
Shankar et al. [50], 2021	18-channel	NR	BR and RP	No	①Children's Hospital Boston;<br>②Bonn University	Bonn<br>training dataset :6400<br>testing dataset :1600<br>CHB<br>training dataset :512069<br>testing dataset :128017	CHB-MIT:2010<br>Bonn :2001	Yes
Sahani et al. [48], 2021	Single-channel	Yes	RDCNN-MKRVFLN	NR	①NSC<br>②Children's Hospital Boston;<br>③Bonn University	NSC<br>training dataset :4500<br>testing dataset :2700<br>validation dataset:1800<br>Bonn	NSC:2016<br>CHB-MIT:2010<br>Bonn :2001	Yes

						training dataset :4000<br>testing dataset :2400<br>validation dataset :1600<br>CHB<br>training dataset :320043<br>testing dataset :192026<br>validation dataset :128017		
Praveena et al. [44], 2021	NR	NR	RICA-LSTM	No	SWEC-ETHZ iEEG database	training dataset :7387200<br>testing dataset :1846800	2018	Yes
Nasiri et al. [42], 2021	23-channel	NR	Generalizable Seizure Detection Model	No	Children’s Hospital Boston	training dataset :640086<br>testing dataset :640086	2010	Yes
Jose et al. [32], 2021	NR	NR	Adaptive Rag-ROA-Deep SAE	No	①TUEP dataset;<br>②Children’s Hospital Boston	TUEP<br>training dataset :878400<br>testing dataset :878400<br>CHB<br>training dataset :640086<br>testing dataset :640086	TUEP :2007-2013<br>CHB :2010	Yes
Chakrabarti et al. [22], 2021	23-channel	NR	LSTM	No	①Children’s Hospital Boston②Sleep-Wake-Epilepsy-Center of the University Department of Neurology at the Inselspital Bern dataset	CHB<br>training dataset :320043<br>testing dataset :320043<br>validation dataset :64009<br>SWEC-ETHZ<br>training dataset: 417600<br>testing dataset :417600	CHB :2010<br>SWEC-ETHZ :2007-2013	Yes

Glory et al. [27], 2021	Single-channel<br>128-channel	NR	AHW-BGOA-DNN	Yes	①Children's Hospital Boston;<br>②Bonn University	validation dataset :2835200<br>Bonn<br>training dataset :5760<br>testing dataset :1440<br>validation dataset :1440<br>CHB<br>training dataset :512069<br>testing dataset :128017<br>validation dataset :128017	CHB-MIT:2010<br>Bonn :2001	Yes
Liu et al. [37], 2020	Single-channel	NR	BiOP	No	①Bonn University<br>②Neurology and Sleep Centre, Hauz Khas, New Delhi.	Bonn<br>training dataset:6400<br>testing dataset:1600<br>NSC<br>training set :7200<br>testing dataset :1800	Bonn 2001<br>NSC 2016	Yes
Liu et al. [39], 2020	128-channel<br>3-channel<br>6-channel	NR	S-transform，CNN	Yes	Epilepsy Center of the University Hospital of Freiburg,Germany	training dataset :1782648<br>testing dataset :2593908<br>validation dataset :198072	2015	Yes
Li et al. [33], 2020	5-channel	Yes	CE-stSENet	Yes	Children's Hospital Boston;	training dataset :640086<br>testing dataset :640086	2010	Yes
Iešmantas et al. [29], 2020	10-channel	NR	NR	No	Temple University Hospital EEG Corpus	training dataset :878400<br>validation dataset :878400	2007-2013	Yes
Geng et al. [26], 2020	3-channel	Yes	BiLSTM with S-transform	Yes	Epilepsy Center of the University Hospital of Freiburg,Germany	training dataset :2593908<br>validation dataset :2593908	2015	Yes
Bari et al. [20], 2020	Single-chan	Yes	CEEMDAN	Yes	Bonn University	training dataset :8000	2001	Yes

	nel					testing dataset :8000		
Abiyev et al. [19], 2020	128-channel	NR	Deep CNN	Yes	①Children's Hospital Boston;<br>②Bonn University	Bonn<br>training dataset :7200<br>testing dataset :800<br>validation dataset :240<br>CHB<br>training dataset :576077<br>testing dataset :64009<br>validation dataset :19203	CHB-MIT:2010<br>Bonn :2001	Yes
Yu et al. [68], 2019	Single-chan<br>nel<br>128-channel	Yes	R-ProCRC	Yes	①Bonn University<br>②Epilepsy Center of the University Hospital of Freiburg,Germany	Bonn<br>training dataset :6400<br>testing dataset :1600<br>NSC<br>training dataset :1604160<br>testing dataset :401040	Bonn :2001<br>Freiburg :2015	Yes
Lin et al. [36], 2019	Single-chan<br>nel<br>128-channel	NR	CC<br>algorithm	No	Bonn University	training dataset:7600<br>testing dataset :400	2001	Yes
Jiang et al. [30], 2019	Single-chan<br>nel<br>23-channel	NR	RR-DTDWT	Yes	①Children's Hospital Boston;<br>②Bonn University	Bonn<br>training dataset :40000<br>testing dataset :40000<br>CHB<br>training dataset :640086<br>testing dataset :640086	CHB-MIT:2010<br>Bonn :2001	Yes
Abdelhameed et al. [17], 2019	23-channel	Yes	VAE	Yes	①Children's Hospital Boston;	Bonn<br>training dataset :7200	CHB-MIT:2010<br>Bonn :2001	Yes

					②Bonn University	validation dataset :800<br>testing dataset :800<br>CHB<br>training dataset: 576077<br>validation dataset :64009<br>testing dataset :64009		
Yuan et al. [69], 2018	23-channel	NR	BLDA	Yes	Children’s Hospital Boston	testing dataset :640086<br>training dataset :640086	2010	Yes
Bhattacharyya et al. [21], 2017	23-channel	Yes	EWT	Yes	Children’s Hospital Boston	training dataset :320043<br>testing dataset :320043	2010	Yes
Zabihi et al. [71], 2016	23-channel	Yes	NR	No	Children’s Hospital Boston	training dataset :320043<br>testing dataset :320043	2010	Yes
Xiong et al. [66], 2023	6-channel	NR	MVMD	NR	①Children’s Hospital Boston<br>②Siena Scalp dataset of the Unit of Neurology and Neurophysiology of the University	CHB-MIT<br>training dataset :480065<br>testing dataset :160022<br>Siena Scalp<br>training dataset :345600<br>testing dataset :115200	CHB-MIT:2010<br>Siena Scalp :2020	Yes
Visalini et al. [61], 2023	23-channel	NR	DBN	Yes	Helsinki University Hospital, Finland,	training dataset :216840<br>testing dataset :216840	2010 and 2014	Yes
Dong et al. [24], 2023	20-channel	NR	WPD-RF	Yes	①Children’s Hospital Boston	training dataset :511371<br>testing dataset :27787	2010	Yes
Xiong et al. [65], 2022	20-channel	NR	NR	Yes	①Children’s Hospital Boston<br>②Siena Scalp dataset of the Unit of Neurology and Neurophysiology of the	CHB-MIT<br>training dataset :448060<br>testing dataset :192026<br>Siena Scalp	CHB-MIT:2010<br>Siena Scalp :2020	Yes

					University	training dataset :322560<br>testing dataset :138240		
Razi et al. [47], 2022	NR	NR	FoM_SD	No	Sleep-Wake-Epilepsy-Center of the University Department of Neurology at the Inselspital Bern dataset	training dataset :3029760<br>testing dataset :574400	2018	Yes
Liu et al. [38], 2022	6-channel	NR	VMFs-LECM	Yes	①Bonn University<br>②Epilepsy Center of the University Hospital of Freiburg,Germany	Bonn<br>training dataset :36000<br>testing dataset :4000<br>Freiburg<br>training dataset :2334517<br>testing dataset :259391	Bonn :2001<br>Freiburg :2015	Yes
Solaija et al. [55], 2018	18-channel<br>22-channel	NR	DMD	Yes	①Children's Hospital Boston	CHB-MIT<br>training dataset :1709500<br>testing dataset :1740167	2012	Yes
Vidyaratne et al. [60], 2017	NR	Yes	NR	Yes	①Bonn University<br>②Children's Hospital Boston	Bonn<br>training dataset :32000<br>testing dataset :8000<br>validation dataset :8000<br>CHB-MIT<br>training dataset :508799<br>testing dataset :127200<br>validation dataset :127200	Bonn :2001<br>CHB-MIT:2010	Yes
Zhang et al. [72], 2015	128-channel<br>6-channel	NR	NR	Yes	the Epilepsy Center of the University Hospital of Freiburg,Germany	training dataset :1089720<br>testing dataset :1089720	2015	Yes
