# Supplementary material for: Epileptic Seizure Detection Using Machine Learning: A Systematic Review and Meta-Analysis
Source: Brain Sci. 2025 Jun 12;15(6):634. doi: 10.3390/brainsci15060634 (PMC12190198; doi:10.3390/brainsci15060634)
Supplement: Supplementary file 1 [file brainsci-15-00634-s001.zip › Table S3.pdf]

Table S3. Design and basic demographics.

Author [ref], year	Participants			N
	**Inclusion criteria**	**Exclusion criteria**	**Labels**	
Sun et al. [57], 2024	We could not read some of the channels in chb15 and chb16.	data from these two patients were removed.	epileptic seizure	CHB-MIT:22<br>Bonn : 10
Li et al. [34], 2024	NR	NR	epileptic seizure	Bonn : 10
Jibon et al. [31], 2024	NR	NR	epileptic seizure	CHB-MIT :22<br>TUH :1385
Chung et al. [23], 2024	As they reviewed only electrographic seizures without video data, seizure locations of six cases could not be identified (chb12, chb14, chb16, chb18, chb20, and chb21), and those of four cases were not close to Fp1-F3, Fp2-F4, P7-O1, or P8-O2 (chb06, chb09, chb13, and chb19). Therefore,	they selected 13 cases whose seizure locations were identifiable by four or one of the four channels.	epileptic seizure	CHB-MIT:13
Abdulwahhab et al. [18], 2024	For the purpose of epileptic seizure detection in this study, the raw EEG recordings from 18 specific channels were utilized.	NR	epileptic seizure	Bonn :10<br>CHB-MIT:22
Rani et al. [46], 2024	NR	NR	epileptic seizure	Bonn :10
Zhao et al. [73], 2023	The dataset is recorded at 512 Hz sampling rate. We select five men (PN00, PN06, PN10, PN12, PN14) and five women (PN05, PN07, PN09, PN11, PN13) for experiments.	NR	epileptic seizure	CHB-MIT:22
Wang et al. [63], 2023	Since most seizure activity occurs in this 0.5–40 Hz frequency range (Zarei and Asl, 2021), band-pass filtering of 0.5–40 Hz is carried out in this study for the two databases mentioned above(the CHB-MIT Database，the Siena Scalp Database).	NR	epileptic seizure<br>Seizure minutes	CHB-MIT:24<br>Bonn :10<br>Siena Scalp :14
Srinivasan et al. [56], 2023	Chb12 and Chb13’s seizures were lef out due to changes in	Chb16’s seizures lasted less than	epileptic seizure	CHB-MIT:16

	channel names and electrode placements. Four patients aged 16 and up (Chb04, Chb15, Chb18, and Chb19) were excluded since the focus is on detecting seizures in young children.	10 s so none were considered for testing.		
Si et al. [53], 2023	NR	We excluded iEEG data containing fewer than three seizures during the recording period.	epileptic seizure	SWEC-ETHZ:13
Shanmugam et al. [51], 2023	NR	NR	epileptic seizure	Bonn :5<br>NSC:10
Reddy et al. [49], 2023	All these subjects are aged between 23 and 55 years	NR	epileptic seizure	Bonn :5
Prasanna et al. [44], 2023	Recordings from 23 juvenile epilepsy patients with the condition are included in the collection	NR	epileptic seizure	CHB-MIT:23
Poorani et al. [43], 2023	Totally 23 patients data are available in the dataset. Due to the complexity of the EEG signals	data of only frst nine patients are considered in this experiment.	epileptic seizure	CHB-MIT:9
Mir et al. [41], 2023	NR	NR	epileptic seizure	CHB-MIT:35
Huang et al. [28], 2023	If a patient's seizure lasts less than 400 s, over-sampling is utilized to ensure sufficient samples,patients with drug-resistant epilepsy who were evaluated for epilepsy surgery at the Sleep-Wake-Epilepsy Center (SWEC) of the Department of Neurology at Bern Hospital University	NR	epileptic seizure	Bonn :10<br>SWEC-ETHZ:22
Zhao et al. [74], 2022	The dataset was collected under the condition that patients have clear epileptic seizures after stopping anti-epileptic treatments.	NR	epileptic seizure	CHB-MIT :23
Yuan et al. [70], 2022	NR	NR	epileptic seizure	Freiburg :21
Yan et al. [67], 2022	NR	NR	epileptic seizure	CHB-MIT:24;<br>Bonn :10
Sun et al. [58], 2022	We only considered subjects with no less than three leading	we excluded five pa tients: three	epileptic seizure	SWEC-ETHZ:13

	seizures.	patients had no seizures during SEEG, and two had brain disorders other than epilepsy.		
Sivasaravanababu et al. [54], 2022	From the initially selected EEG signals of patients as in 1, 3, 6, 7 and 10	EEG of patient 6 was rejected since almost all of their seizures lasted for time duration of less than 16s.	epileptic seizure	CHB-MIT :23
Shoeibi et al. [52], 2022	At least 2 and at most 5 epileptic seizures were observed in each subject	NR	epileptic seizure	Freiburg :21<br>Bonn :10
Lian et al. [35], 2022	To guarantee the same resolution of the input graphs,only the first 19 EEG channels were selected for every subject.only 100 epochs with seizures were used in the following experiments.	NR	epileptic seizure	CHB-MIT :23
Duan et al. [25], 2022	NR	NR	epileptic seizure	Bonn :11<br>CHB-MIT :23
Maheshwari et al. [40], 2022	The window has been moved by ten samples with an overlap of 246 samples. If the overlap is too small, we will get too much fluctuations in the distance trajectories, whereas if the overlap is too large, though the distance trajectories will be smooth, we can miss out on salient transitions. Based on these considerations, we found the overlap of ten samples to be optimum	NR	epileptic seizure	CHB-MIT:23
Woodbright et al. [64], 2021	NR	NR	epileptic seizure	Bonn :10
Wang et al. [62], 2021	A concatenated seizure or a raw seizure lasting more than 10 s was chosen, and so seizures which lasted less than 10 s were not considered.	NR	epileptic seizure	CHB-MIT:24<br>SWEC-ETHZ :18
Thara et al. [59], 2021	NR	NR	epileptic seizure	Bonn :10
Shankar et al. [50], 2021	The Pict and Inict segments are chosen from one and four	NR	epileptic seizure	Bonn :10

	hours before starting of ictal event respectively , which keeping the segment length of ictal event for each subject . Based on this condition, seven subjects (chb02, chb06, chb14, chb16, chb20, chb21, and chb22) having common 18 channels			CHB-MIT :23
Sahani et al. [48], 2021	NR	In our experiment, three .edf files (i.e., chb-12,-27 to 29) are not considered because of montage paradox compared to other.edf files.	epileptic seizure	Bonn :10<br>NSC:10<br>CHB-MIT :23
Praveena et al. [44], 2021	NR	NR	epileptic seizure	SWEC-ETHZ:18
Nasiri et al. [42], 2021	NR	NR	epileptic seizure	CHB-MIT :23
Jose et al. [32], 2021	NR	NR	epileptic seizure	TUEP :200<br>CHB-MIT :23
Chakrabarti et al. [22], 2021	NR	NR	epileptic seizure	CHB-MIT:23<br>SWEC:16
Glory et al. [27], 2021	The band-pass filter is set to filter out the EEG signal of 0.5–150 Hz. The band-pass filter is set to filter out the EEG signal of 0.53-40 Hz. The continuous recordings were obtained with 16-bit resolution, a sampling frequency of 256 Hz and the electrodes are placed in international 10-20 electrode position system	NR	epileptic seizure	Bonn :10<br>CHB-MIT:23
Liu et al. [37], 2020	NR	NR	epileptic seizure	Bonn :10<br>NSC:10<br>THUZ:190
Liu et al. [39], 2020	NR	NR	epileptic seizure	Freiburg:21
Li et al. [33], 2020	EEG recordings which contain 21 common channels and at	We fail to read some channel data	epileptic seizure	CHB-MIT:24

	least one seizure event have been considered	for patients 6, 12 and 16, thus these three patients have been removed		
Iešmantas et al. [29], 2020	NR	NR	epileptic seizure	TUH:10874
Geng et al. [26], 2020	Patients suffering from medically intractable epilepsy	Note that patient 10 is not considered with the reason of electrode box disconnection	epileptic seizure	Freiburg:21
Bari et al. [20], 2020	All of whom had achieved complete seizure control after resection of the detected epileptogenic zone-one of the hippocam pal formations.	In this work, we have used only set N, F and S.	epileptic seizure	Bonn:10
Abiyev et al. [19], 2020	NR	NR	epileptic seizure	Bonn :10<br>CHB-MIT :23
Yu et al. [68], 2019	NR	For each patient, nomore than three seizure events are selected according to the time order (except patients 5, 15, and 19) and the twice num ber of non-seizure data are selected for training.	epileptic seizure	Bonn :10<br>Freiburg :21
Lin et al. [36], 2019	NR	NR	epileptic seizure	Bonn :10
Jiang et al. [30], 2019	NR	NR	epileptic seizure	Bonn:10<br>CHB-MIT:23
Abdelhameed et al. [17], 2019	Among the originally selected patients (1, 3,6, 7 and 10)	as in patient 6 was excluded because most of its seizures were shorter than 16 seconds.	epileptic seizure	Bonn :10
Yuan et al. [69], 2018	For every patient,there were 18–145 h EEG recordings including periods of wake and sleep, and at least three seizures.	NR	epileptic seizure	CHB-MIT:23

Bhattacharyya et al. [21], 2017	NR	We failed to read the EEG signals for the patient 12, thus in this study patient 12 has not been considered.	epileptic seizure	CHB-MIT:23
Zabihi et al. [71], 2016	NR	In addition, patient 15 was excluded from our analysis because we failed to read the EEG data of this patient.	epileptic seizure	CHB-MIT:23
Xiong et al. [66], 2023	NR	NR	epileptic seizure	CHB-MIT:23<br>Siena Scalp :14
Visalini et al. [61], 2023	NR	NR	neonatal seizure detection	Helsinki :39
Dong et al. [24], 2023	In order to filter the low-frequency and high-frequency artifact interference components in the EEG signals, the band-pass filter of the system is set to 1 Hz to 50 Hz.	NR	Event-based real-time seizure detection epileptic seizure	CHB-MIT:24
Xiong et al. [65], 2022	NR	NR	epileptic seizure	CHB-MIT:24<br>Siena Scalp :14
Razi et al. [47], 2022	NR	NR	epileptic seizure	SWEC-ETHZ:18
Liu et al. [38], 2022	NR	NR	epileptic seizure	Bonn :10<br>Freiburg :21
Solaija et al. [55], 2018	NR	NR	epileptic seizure	CHB-MIT:25
Vidyaratne et al. [60], 2017	Dataset A consists of long-term bipolar referenced EEG recordings from<br>pediatric patients with intractable seizures.The seizure duration is presented for individual	The EEG recordings of 'chb16' are excluded from the analysis due to the unusually short seizure duration with an average ictal	epileptic seizure	Bonn :20<br>CHB-MIT:24

	seizure events if the total number of events per patient is less than or equal to 4. Otherwise, the mean duration with standard deviation (stdev) is presented	length of mere 8.6 s.		
Zhang et al. [72], 2015	NR	NR	epileptic seizure	Freiburg :21
