# Supplementary figures and images for: Epileptic Seizure Detection Using Machine Learning: A Systematic Review and Meta-Analysis
Source: Brain Sci. 2025 Jun 12;15(6):634. doi: 10.3390/brainsci15060634 (PMC12190198; doi:10.3390/brainsci15060634)

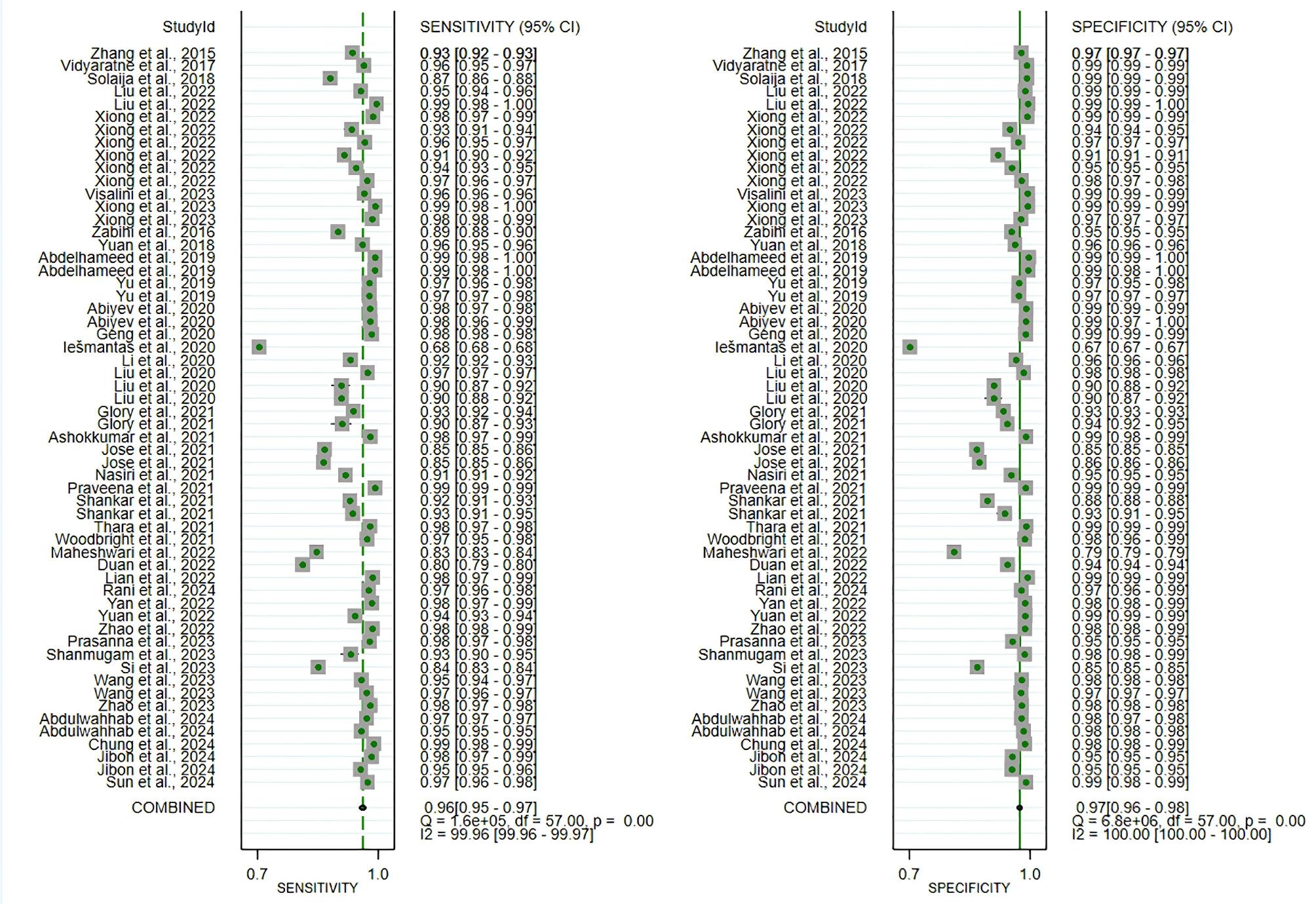

Supplement: Supplementary file 1 [file brainsci-15-00634-s001.zip › Figure S1. Forest plots for all studies.jpg]

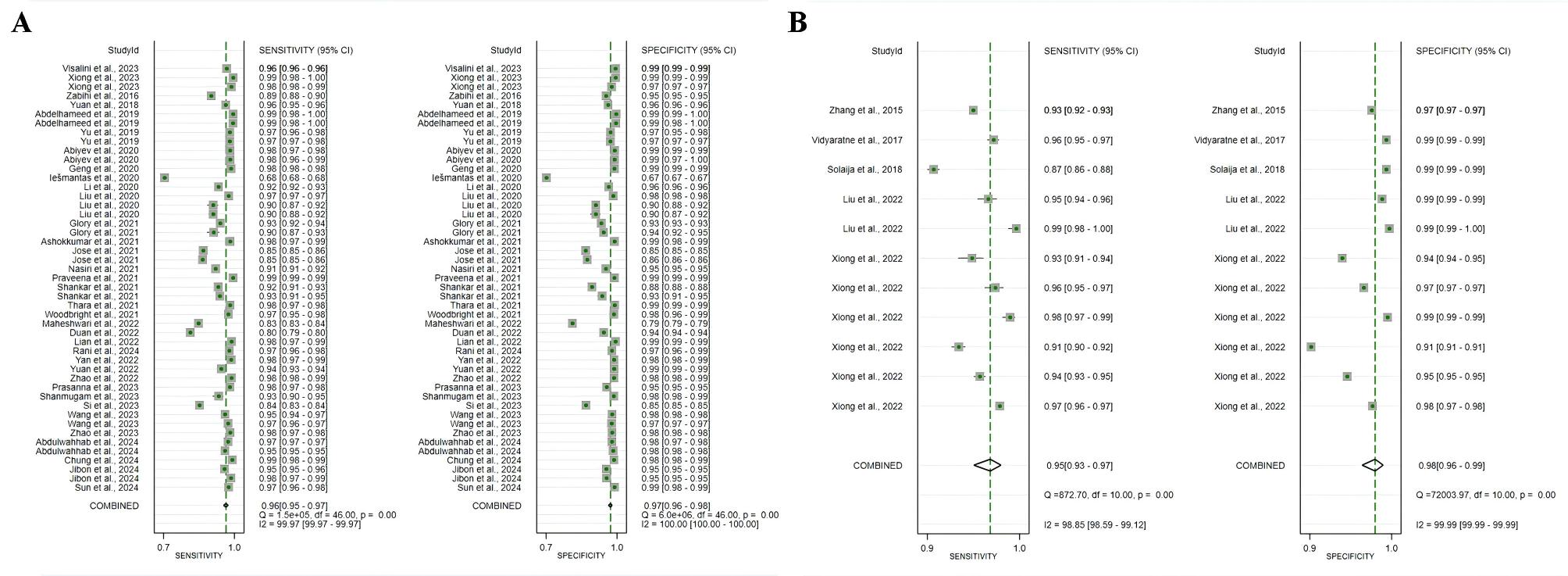

Supplement: Supplementary file 1 [file brainsci-15-00634-s001.zip › Figure S2. Forest maps of different model classifications (DL(A), ML(B)).jpg]

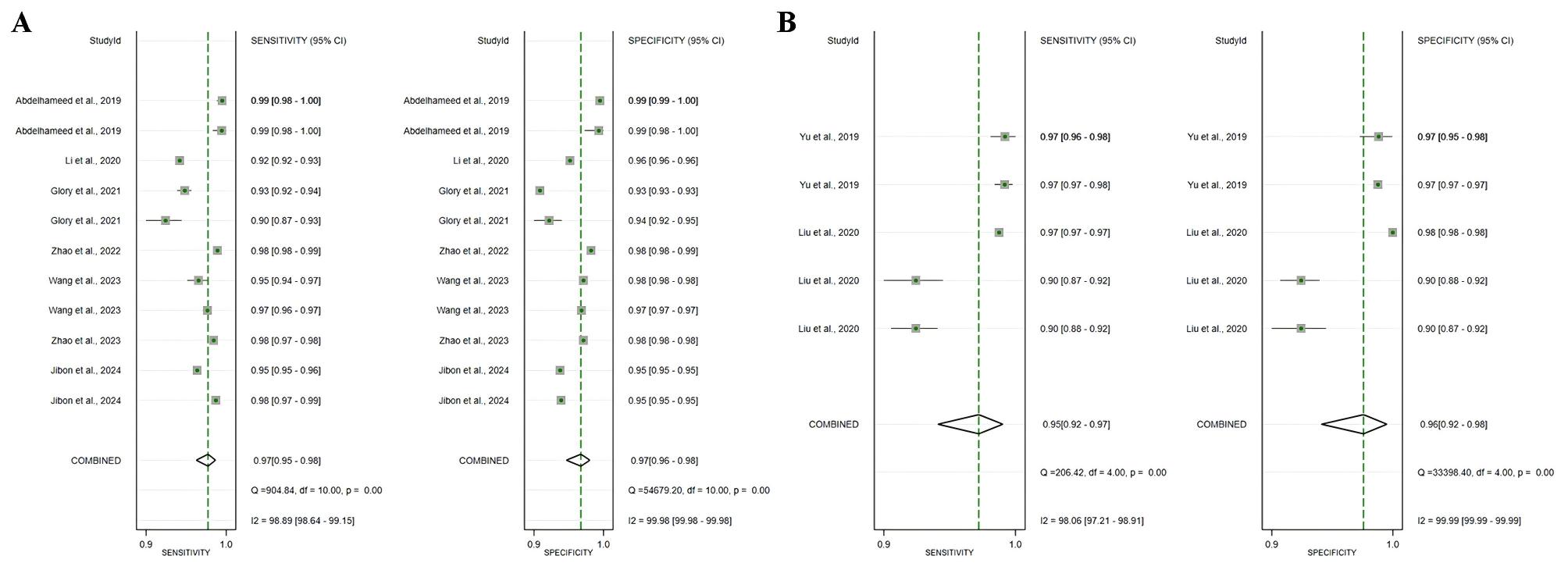

Supplement: Supplementary file 1 [file brainsci-15-00634-s001.zip › Figure S3. Different (feature type) data preprocessing methods (band-pass filtering (A) or DWT forest graph (B)).jpg]

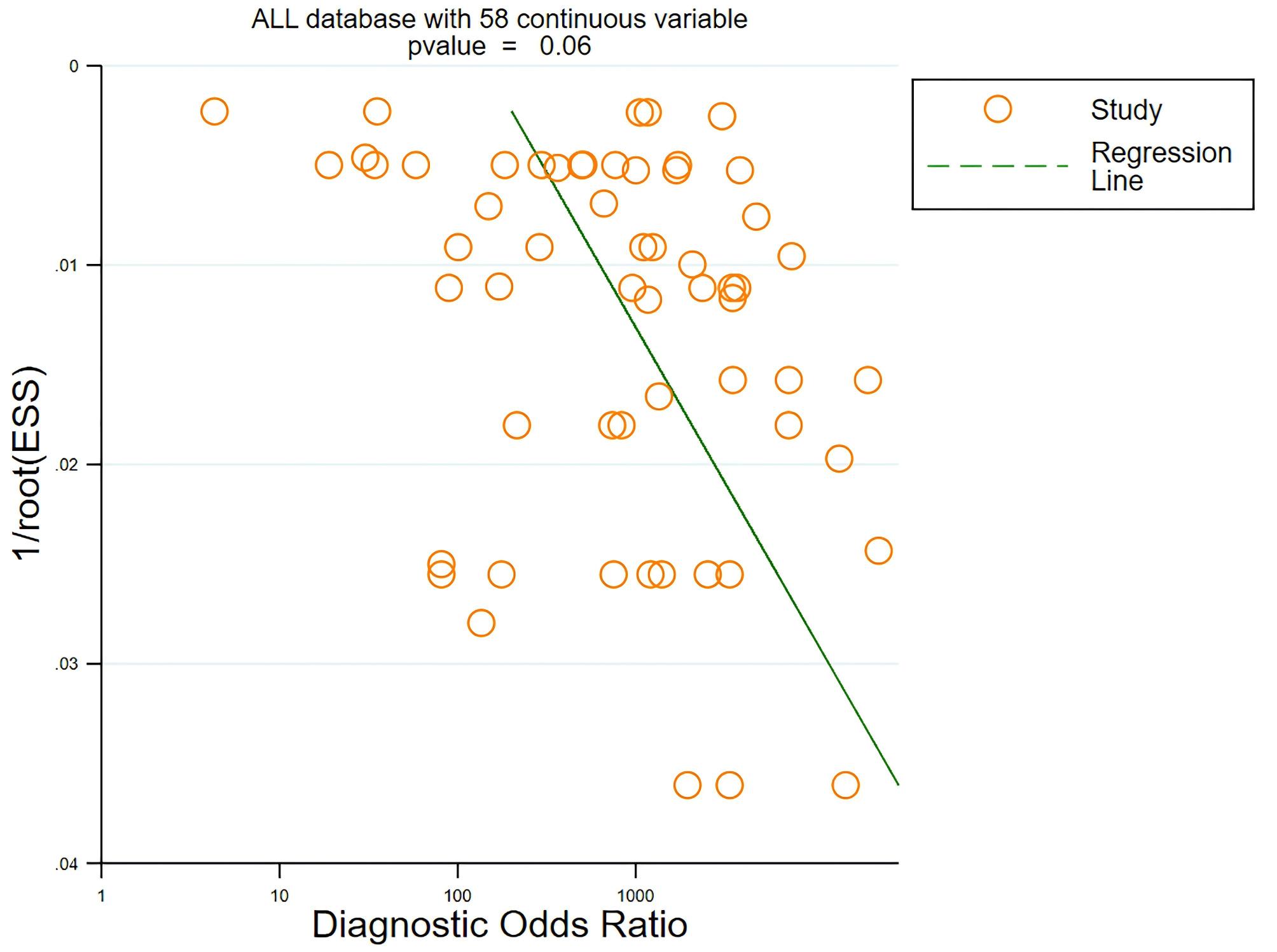

Supplement: Supplementary file 1 [file brainsci-15-00634-s001.zip › Figure S5. Publication bias.jpg]
